# Endogenous Cytokinin Overproduction Modulates ROS Homeostasis and Decreases Salt Stress Resistance in *Arabidopsis Thaliana*

**DOI:** 10.3389/fpls.2015.01004

**Published:** 2015-11-19

**Authors:** Yanping Wang, Wenzhong Shen, Zhulong Chan, Yan Wu

**Affiliations:** ^1^State Key Laboratory of Hybrid Rice, College of Life Sciences, Wuhan UniversityWuhan, China; ^2^Key Laboratory of Plant Germplasm Enhancement and Specialty Agriculture, Wuhan Botanical Garden, Chinese Academy of SciencesWuhan, China

**Keywords:** cytokinin overproduction, *AtIPT8*, ROS homeostasis, salt stress, chlorophyll, transcriptomic analysis

## Abstract

Cytokinins in plants are crucial for numerous biological processes, including seed germination, cell division and differentiation, floral initiation and adaptation to abiotic stresses. The salt stress can promote reactive oxygen species (ROS) production in plants which are highly toxic and ultimately results in oxidative stress. However, the correlation between endogenous cytokinin production and ROS homeostasis in responding to salt stress is poorly understood. In this study, we analyzed the correlation of overexpressing the cytokinin biosynthetic gene *AtIPT8* (adenosine phosphate-isopentenyl transferase 8) and the response of salt stress in *Arabidopsis*. Overproduction of cytokinins, which was resulted by the inducible overexpression of *AtIPT8*, significantly inhibited the primary root growth and true leaf emergence, especially under the conditions of exogenous salt, glucose and mannitol treatments. Upon cytokinin overproduction, the salt stress resistance was declined, and resulted in less survival rates and chlorophyll content. Interestingly, ROS production was obviously increased with the salt treatment, accompanied by endogenously overproduced cytokinins. The activities of catalase (CAT) and superoxide dismutase (SOD), which are responsible for scavenging ROS, were also affected. Transcription profiling revealed that the differential expressions of ROS-producing and scavenging related genes, the photosynthesis-related genes and stress responsive genes were existed in transgenic plants of overproducing cytokinins. Our results suggested that broken in the homeostasis of cytokinins in plant cells could modulate the salt stress responses through a ROS-mediated regulation in *Arabidopsis*.

## Introduction

Cytokinins play important and complex roles in plant growth and abiotic stress responses (Wang et al., 2011; Ha et al., 2012; Hwang et al., 2012; Zwack and Rashotte, 2015). Numerous evidences indicate that cytokinins have both positive and negative effects on stress tolerance. Many studies have reported that, in response to extended stress, the concentrations of cytokinins were decreased in plants (Kudoyarova et al., 2007; Ghanem et al., 2008; Merewitz et al., 2011; Nishiyama et al., 2011). Contrarily, both short-term and sustained increase in cytokinin levels may also occur in plants while encountering severe stress conditions (Pospisilova et al., 2005; Alvarez et al., 2008; Dobra et al., 2010). Cytokinin biosynthesis genes *IPTs* (adenosine phosphate-isopentenyl transferases) can be up-regulated by NaCl treatment, and the deficiency in cytokinin biosynthesis may result in a strong salt-tolerant phenotype (Nishiyama et al., 2011). Many studies have examined the effects of exogenous cytokinin applications in abiotic stress responses. Exogenously supplied cytokinins not only can improve salt tolerance in young wheat seedlings, but also can result in more susceptible phenotype to the salt treatment in beans (Kirkham et al., 1974; Abdullah and Ahmad, 1990). After cytokinin application, the *Arabidopsis* plants are of higher survival ability when they are exposed to freezing or dehydrated conditions (Jones et al., 2010; Kang et al., 2012). The effects of changed endogenous cytokinin levels in transgenic plants overexpressing cytokinin biosynthesis genes (*IPTs*), or cytokinin degraded genes (*CKXs*), are demonstrated. Overproduction of endogenous cytokinins enhances drought stress tolerance. However, decrease in cytokinin levels produce a positive consequence in drought tolerance (Rivero et al., 2007; Werner et al., 2010; Qin et al., 2011; Macková et al., 2013).

The components of cytokinin signaling also play complex roles in responses to abiotic stresses. For instance, *Arabidopsis* AHK1, the histidine kinase 1 of cytokinin signaling, plays as a positive regulator in the responses of drought and salt stresses. The loss-of-function mutations, such as *ahk2, ahk3*, and *ahk2 ahk3* are of strong tolerance to drought and salt stresses (Tran et al., 2007; Wohlbach et al., 2008; Kumar et al., 2013). AHPs (histidine phosphotransfer proteins) are involved in regulating the responses to drought stress in a negative and redundant manner (Hutchison et al., 2006; Hwang et al., 2012; Nishiyama et al., 2013). The resistant to salt stress phenotype is reported in studying the quadruple loss-of-function mutant *arr3arr4arr5arr6* (Mason et al., 2010). Collectively, all these studies suggest the impact of cytokinin metabolism and signaling in the stress responses in intricate manners.

The reactive oxygen species (ROS) such as hydrogen peroxide (H_2_O_2_), superoxide radical (O_2_^-^), and hydroxyl radical (OH^-^), all can be induced by drought, salt, and low temperature conditions (Sharma et al., 2012; Choudhury et al., 2013; Petrov et al., 2015). To detoxify, plants have evolved ROS scavenging systems that involve in enzymic and non-enzymic antioxidants. The major antioxidant enzymes include superoxide dismutase (SOD), ascorbate peroxidase (APX), catalase (CAT) and glutathione peroxidase (GPX). SOD converts superoxide into H_2_O_2_, while APX, GPX, and CAT detoxify H_2_O_2_ (Mittler, 2002; Apel and Hirt, 2004; Das and Roychoudhury, 2014). The cross-talk between the cytokinin signaling and ROS production and scavenging systems is demonstrated in *Arabidopsis*. In cytokinin-deficient mutant *ipt1,3,5,7*, the genes involving in ROS breakdown are greatly affected (Nishiyama et al., 2012). The treatment of N(6)-benzyladenine (6-BA) induces massive production of ROS, eventually, results in a loss of cell viability in tobacco BY-2 cells (Mlejnek et al., 2003). Exogenous applications of cytokinins lead to increasing in APX and CAT activities during dark-induced senescence (Zavaleta-Mancera et al., 2007), as well as raise of SOD and CAT activities after heat stress (Liu and Huang, 2002). In overexpressing *CKX* transgenic *Arabidopsis* lines, declined cytokinin levels may cause alterations in activities of antioxidants, while responding to abiotic stresses (Mýtinová et al., 2010; Lubovská et al., 2014). Hence, the impact of cytokinins on ROS homeostasis in plants responding to environmental stresses is imperative.

To in-depth study the correlation between endogenous cytokinin levels and ROS homeostasis in plants responding to abiotic stresses especially to the salt stress, we analyzed the inducible transgenic line overexpressing *AtIPT8*, a cytokinin biosynthesis gene. The results indicated that endogenous cytokinin overproduction, which was promoted by *AtIPT8* overexpression, resulted in enhanced-sensitive phenotype to the salt treatment. Dependent on salt treatment, the ROS contents were strongly increased in plants of overproducing cytokinin; and, the activities of antioxidants and the contents of total chlorophyll were significantly declined with comparing to those in the wild-type (Col). Moreover, many genes involving in photosynthesis and abiotic stress responses were differentially expressed in plants of overexpressing *AtIPT8*. In this study, we provided evidences in that overproduction of endogenous cytokinin could decrease salt resistance, through modulating endogenous ROS homeostasis in *Arabidopsis*.

## Materials and Methods

### Plant Materials and Growth Conditions

*Arabidopsis thaliana* ecotype Columbia (Col) was used in this study as wild-type control. Seeds were surface sterilized and sown on Murashige and Skoog (MS) agar plates containing full-strength MS salts, 0.8% (w/v) agar, and 1% (w/v) sucrose. The 17-β-estradiol (Sigma-Aldrich, E8875) was dissolved in DMSO (Dimethyl Sulfoxide) and used in this study. The seeds were stratified in darkness at 4°C for 4 days and then transferred to growth chamber with 16 h/8 h light/dark cycle at 23°C, or were directly sown in soil after stratification under the same conditions. Overexpressing *AtIPT8* transgenic plants (*OE*) were generated in Col-0 background as described by Wang et al. (2011). The homozygous T4 transgenic lines were used in this study.

### Cytokinins Extraction and Quantification

Cytokinins were extracted and purified from 2 g of 2-week-old seedlings which were induced with 17-β-estradiol (10 μM) for 24 h (Wang et al., 2011). The extraction procedure was performed according to methods described in previous reports (Åstot et al., 2000; Dobrev and Kamínek, 2002; Hoyerová et al., 2006). The internal standards of Deuterium-labeled cytokinin (Olchemim, Czech) were added to the extraction buffer (100 ng per sample). Detection and quantification of cytokinins were performed with HPLC-MS system (Agilent 1200 series HPLC, Agilent Technologies, Palo Alto, CA, USA; AB 3200 Q trap MS/MS, Applied Biosystems, USA).

### Comparisons of Root Growth, Survival Rates and Chlorophyll Contents

To compare the primary root growth under various stress treatments, seeds were respectively sown on MS agar plates supplied with NaCl (100 mM), glucose (300 mM) and D-mannitol (300 mM). 17-β-estradiol (10 μM) or DMSO (mock) was added to the plates. After stratified at 4°C for 4 days, the plates were transferred to growth chamber and placed vertically. The primary root length was measured at the 10 days after transferring. For salt resistance treatment, 5-day-old seedlings grown on MS plates were transferred to fresh salt-containing MS plates, and then calculated the survival rates after 10 days treatment. The seedlings after survival rates calculation were collected and used for chlorophyll contents determination. Total chlorophyll was extracted in 85% acetone as described by Porra et al. (1989). The contents of chlorophyll were determined at settings of 639 nm and 645 nm, respectively with spectrophotometer. All experiments were performed three times independently.

### Determination of ROS Production and Antioxidant Enzymes Activities

Reactive oxygen species production was detected in roots and cotyledons using dichlorofluorescein (DCF; Foreman et al., 2003). The 5-day-old seedlings were treated with 100 mM NaCl plus or minus 17-β-estradiol in plates. After treatment, the seedlings were incubated with 20 μM DCF. To detect the DCF fluorescent signals, images were acquired with confocal laser scanning microscopy (TCS SP8, Leica, Germany) under 488 nm excitation and 525 nm emission. Fluorescence intensity was quantified using LAS AF software. Quantification of H_2_O_2_ content was determined using the method described by Hu et al. (2012). Ten-day-old seedlings were pre-treated with 100 mM NaCl plus or minus 17-β-estradiol in plates. H_2_O_2_ content and activity of antioxidant enzymes were measured after salt treatments. The detailed procedure has been described by Wang et al. (2013).

### Gene Expression Analysis by Microarray and Quantitative Real-time RT-PCR (qRT-PCR)

For microarray analysis, 10-day-old plants of Col and *AtIPT8-OE* were pre-treated with 17-β-estradiol (10 μM) or DMSO for 24 h, respectively. Afterward, the seedlings were collected for total RNA extraction and transcriptomic analysis. The detailed procedure has been described by Wang et al. (2011). To confirm the expression patterns of differentially expressed genes obtained from microarray analysis, qRT-PCR was employed after the seedlings pretreated with or without 17-β-estradiol. Total RNA was extracted using a plant RNA purification kit (Tiangen, catalog number #DP432^1^). Equal amounts of RNA were used for reverse transcription with ReverTra Ace-α-TM (TOYOBO, catalog number FSK-100^2^) according to the manufacturer’s instructions. The primers used in real-time quantitative RT-PCR were designed by web tool^3^. The primers used for qRT-PCR experiment are listed in Supplementary Table S1.

## Results

### Induced-overexpression of *AtIPT8* Resulted in Endogenous Cytokinin Overproduction

**FIGURE 1:**
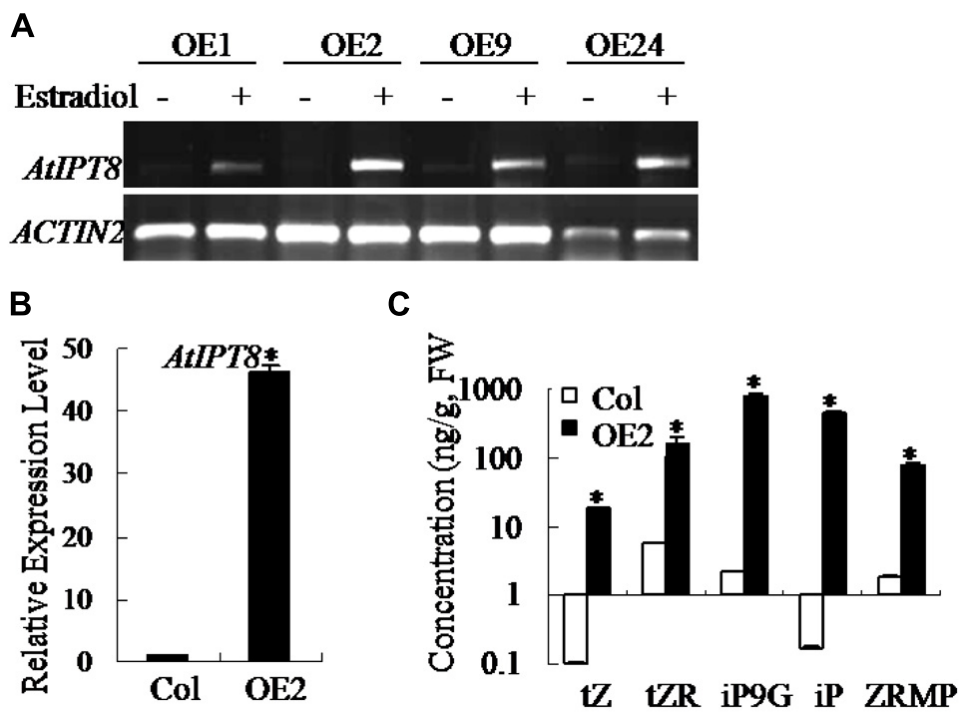
**Overexpression of *AtIPT8* promoted overproduction of endogenous cytokinins in *Arabidopsis*.** Asterisk symbols (^∗^) indicate *p* < 0.05 (Student *t*-test). **(A)** Semi-quantitative RT-PCR to examine the expression levels of *AtIPT8* in independent 17-β-estradiol-inducible transgenic lines. Overexpressing *AtIPT8* transgenic lines (*OE1*, *OE2*, *OE9*, and *OE24*) showed higher expression levels of *AtIPT8* in an estradiol-dependent manner. *ACTIN2* was used as the internal control. Estradiol: 17-β-estradiol (10 μM). **(B)** The *AtIPT8* expression level was more than 40-fold higher in *OE2* plants after 10 μM 17-β-estradiol treatment for 24 h. The values of *Y*-axis indicate means ± SE (standard errors) of three independent experimental repeats. **(C)** The cytokinin varieties in the 2-week-old seedlings of Col and *OE2* were quantified using GC-MS. Seedlings of *OE2* were pre-treated with 10 μM 17-β-estradiol for 24 h. The data represent the means ± SE of two reproducible experiments (fresh weight, FW).

Due to the lethality caused by constitutively overexpressed *AtIPT8* in plants, we generated transgenic plants with estradiol-inducible overexpression of *AtIPT8*, and the line *AtIPT8-OE* was selected (Wang et al., 2011) for further analysis in this study. First, we examined the relative expression levels of *AtIPT8* in transgenic plants using methods of semi-quantitative RT-PCR and qRT-PCR (**Figures 1A,B**). The results showed that expression level of *AtIPT8* gene was induced more than 40-fold higher upon estradiol induction (**Figure 1B**). To examine the effect of *AtIPT8* on the production of endogenous cytokinins, the total cytokinin contents were quantified in plants of Col and *AtIPT8-OE* plants. Upon estradiol induction, the contents of iP and iP9G (iP-type) were increased, more than 100-fold in *AtIPT8-OE* plants than that in Col plants. Moreover, the concentrations of tZ, ZR and ZRMP (Z-type) cytokinins were also elevated more than 10-fold in *AtIPT8-OE* plants (**Figure 1C**). Thus, the quantitative analysis on cytokinin contents indicated that inducer-dependent activation of *AtIPT8* could lead to elevation of cytokinin contents in *Arabidopsis*.

### Endogenous Cytokinin Overproduction Modulated Salt Stress Responses

**FIGURE 2:**
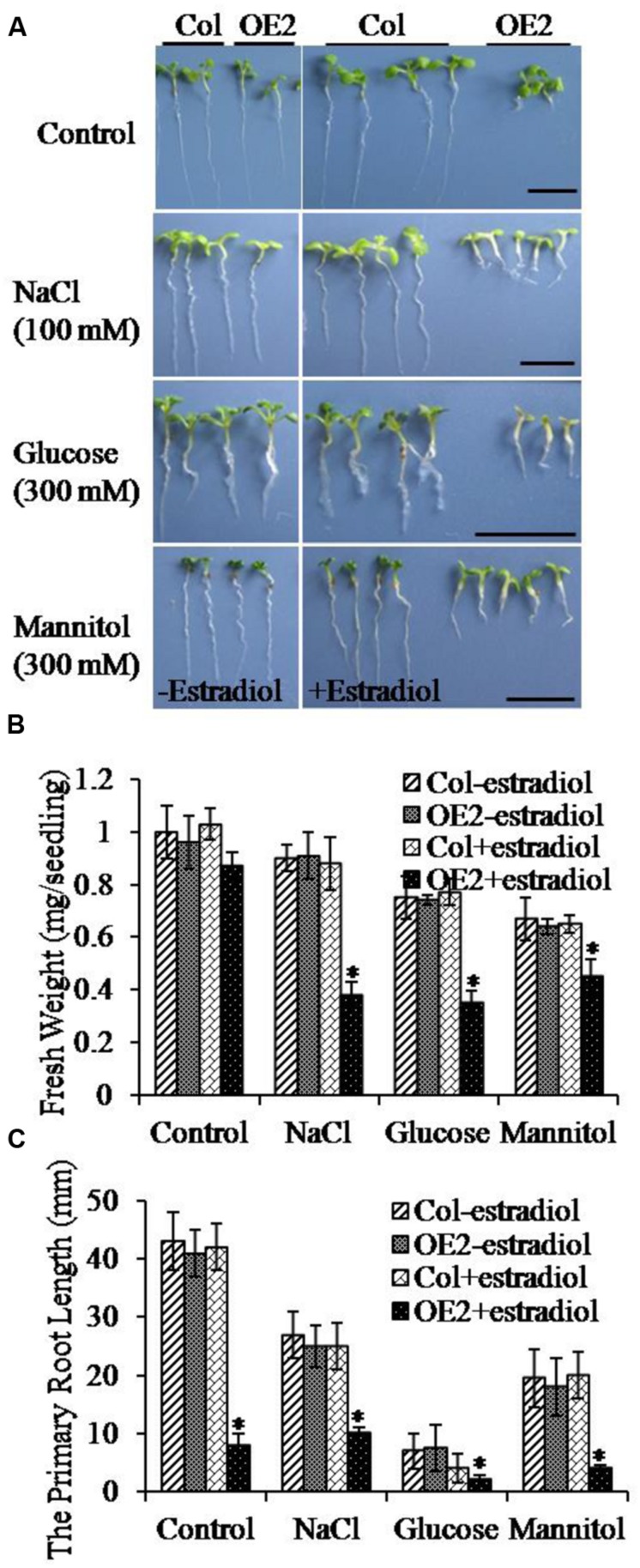
**The growth of cotyledons and primary roots of *OE2* seedlings was altered under various stress conditions.** Asterisk symbols (^∗^) indicate *p* < 0.05 (Student *t*-test). **(A)** The *OE2* seedlings showed hypersensitivity to treatments of NaCl, glucose and mannitol. Seeds were germinated on the indicated plates, and photos were taken after 10 days of seedlings grown on the indicated plates. Bar = 10 mm. Estradiol: 17-β-estradiol (10 μM). **(B)** The fresh weights of Col and *OE2* seedlings were quantified. The values were determined by using the data from three independent experiments (seedling numbers, *n* > 20 per experiment). **(C)** The primary roots lengths of Col and *OE2* seedlings were measured. The values were determined by using the data from three independent experiments (seedling numbers, *n* > 20 per experiment).

To determine whether the endogenous cytokinin levels could affect the abiotic stress responses, we compared the stress resistant phenotypes between Col and *AtIPT8-OE* plants in treatments of NaCl, glucose and mannitol. Seeds were germinated on the freshly prepared MS plates containing NaCl, glucose and mannitol, and then, the fresh weight and primary root length were analyzed after 10 days of treatments. The results showed that cytokinin overproduction limited plant growth (**Figure 2A**, upper panel). Significantly, combining with salt and osmotic stress conditions, the growth of roots and true leaves was inhibited (**Figure 2A**). The fresh weight of plants was obviously decreased in the same stress treatments (**Figure 2B**). Furthermore, we examined the primary root length. The severe effect on root growth was observed with *AtIPT8-OE* plants that were treated by estradiol and NaCl or glucose or mannitol (**Figure 2C**). Interestingly, the most obvious inhibitory effect in the growth of primary roots was showed in the treatment of glucose (300 mM) (**Figure 2C**). The inhibitory effect in primary root growth by cytokinin overproduction was rescued by exogenously addition of auxin (IAA). Application of 2,4-D could trigger more callus generation in *AtIPT8-OE* plants (Supplementary Figure S1). Together, these results suggested that cytokinin overproduction may reduce plant’s tolerance in salt and osmotic stress conditions.

### Endogenous Cytokinin Overproduction Decreased Salt Stress Resistance

**FIGURE 3:**
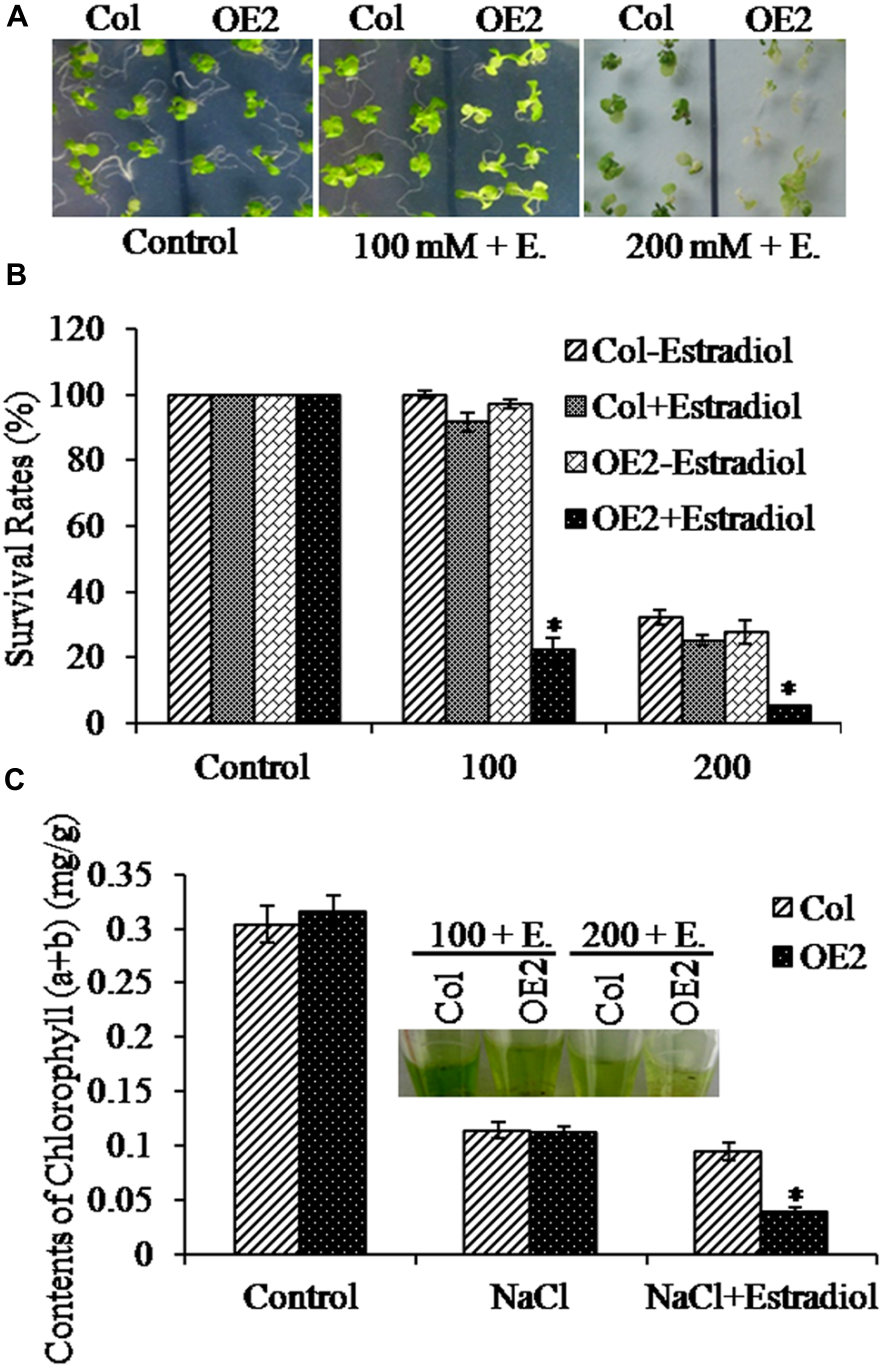
**Analyses of salt stress responses in *OE2* plants.** Asterisk symbols (^∗^) indicate *p* < 0.05 (Student *t*-test). **(A)** Phenotypes of 15-day-old plants of Col and *OE2* on MS plates containing NaCl (100 and 200 mM, respectively). The 5-day-old seedlings grown on MS plates were transferred into the corresponding NaCl-containing plates. Photos were taken after 10 days of treatments. Estradiol: 17-β-estradiol (10 μM). **(B)** The survival rates of Col and *OE2* plants were measured after treated with NaCl (100 and 200 mM, respectively). The data are means ± SE of three independent experiments (*n* > 15 for each repeat). **(C)** The chlorophyll contents of Col and *OE2* plants were measured after treated with NaCl (100 mM) with or without estradiol induction. The data are means ± SE of three independent experiments (*n* > 15 for each repeat).

To further analyze the salt stress response with overproduced cytokinins in plants, we transferred 5-day-old seedlings to the MS plates containing NaCl, and treated for 10 days. We observed that, after estradiol induction, *AtIPT8-OE* plants appeared more sensitive to the treatments of NaCl (**Figure 3A**). Then, we measured the relative survival rates under the conditions of the NaCl treatment. Results showed that, lessen survival rates were scored with *AtIPT8-OE* plants which were induced by estradiol and treated with NaCl; without estradiol induction, no obvious differences, in terms of survival rates, were obtained in plants of Col and *AtIPT8-OE* (**Figure 3B**). The chlorophyll contents are usually used to evaluate the tolerance of plants after stress treatments (Tanaka et al., 2011). Therefore, we measured the total chlorophyll contents in the seedlings. We obtained that decrease in chlorophyll contents caused by NaCl treatments were showing in plants of both Col and *AtIPT8-OE*, more than two-fold decrease in chlorophyll contents was scored with *AtIPT8-OE* plants followed by estradiol induction (**Figure 3C**). Collectively, these results suggested that overproduction of endogenous cytokinins might play a negative effect on surviving in the salinity condition.

### Endogenous Cytokinin Overproduction Increased ROS Accumulation

**FIGURE 4:**
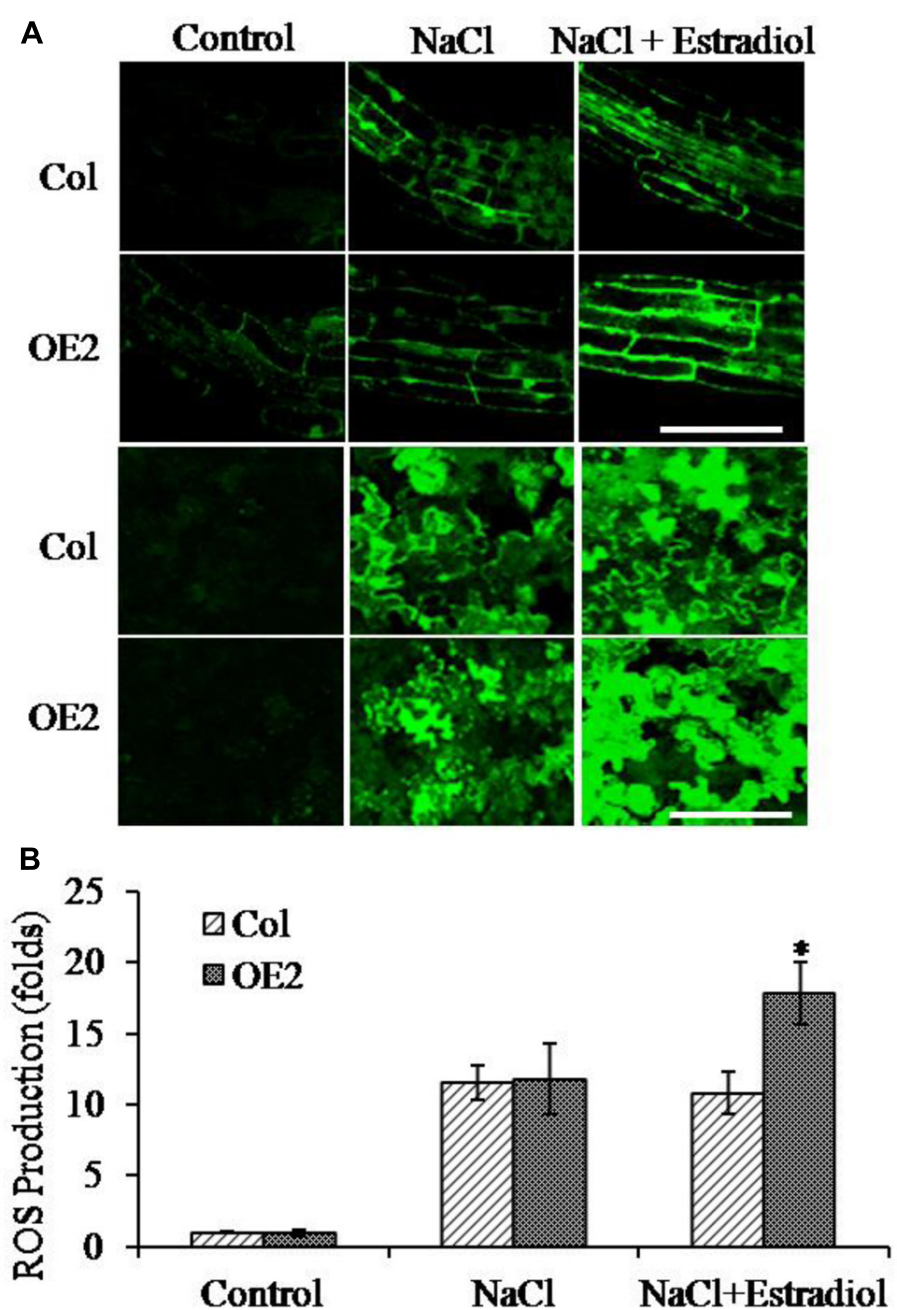
**Reactive oxygen species (ROS) production in roots and cotyledons was analyzed under NaCl treatment.** The salt concentration was 100 mM in this assay. Estradiol, 10 μM. Asterisk symbols (^∗^) indicate *p* < 0.05 (Student *t*-test). **(A)** The fluorescent intensity of DCF was observed with the confocal microscropy. Bar, 50 μm. **(B)** The ROS contents in Col and *OE2* plants were quantified. The data are means ± SE of three independent experiments (*n* = 10). The fluorescent intensity of cotyledons in Col without salt treatment was taken as “1.”

**FIGURE 5:**
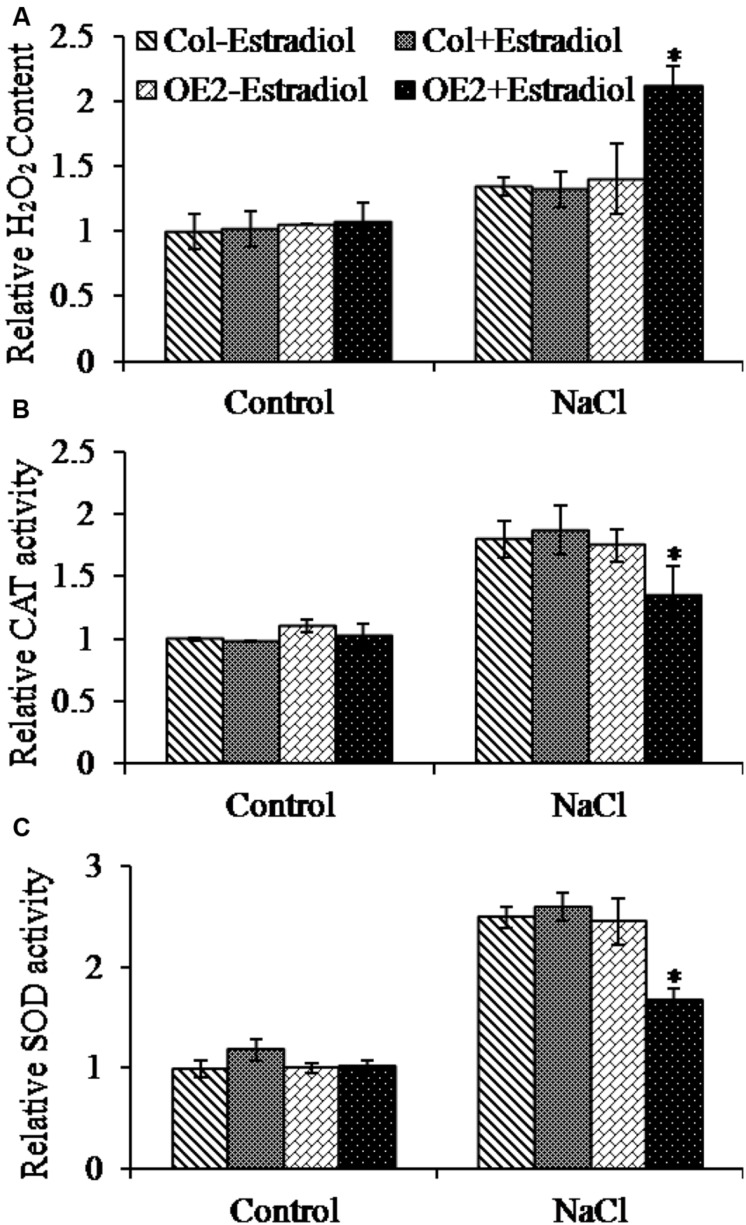
**Determination of relative H_2_O_2_ content and antioxidant enzyme activities in Col and *OE2* plants.** Asterisk symbols (^∗^) indicate *p* < 0.05 (Student *t*-test). The 5-day-old seedlings grown on MS plated were transferred into the indicated treatments for 7 days, and then the seedlings were collected for analyzing H_2_O_2_ and antioxidant enzyme activities (NaCl, 100 mM; Estradiol, 10 μM). Data are means ± SE of three independent measurements. **(A)** Comparison of H_2_O_2_ contents in Col and *OE2* plants after NaCl treatment. **(B)** and **(C)** Changes in CAT and SOD activities of Col and *OE2* were compared.

Salt stress triggers the accumulation of intracellular ROS (Das and Roychoudhury, 2014). To investigate the correlation of cytokinin overproduction and ROS homeostasis in plant cells, we pretreated the seedlings with NaCl and then analyzed the ROS production by quantifying DCF fluorescent intensity. The ROS levels were compared in roots and cotyledons between Col and *AtIPT8-OE* plants. As shown in the results, the NaCl-treatment could promote ROS generation in roots and cotyledons of Col and *AtIPT8-OE* plants (**Figure 4A**). Moreover, the relative salt-induced ROS levels were significantly increased after estradiol-dependent cytokinin overproduction in all of the detected roots and cotyledons (**Figure 4A**). The ROS levels increased more than 10-fold without estradiol induction after salt treatment, however, after estradiol induction, the relative ROS contents were extensively increased about 18-fold in *AtIPT8-OE* plants; whereas there was only 11-fold increased in Col plants when compared with the control treatment (**Figure 4B**). We also examined the effect of exogenous application of 6-BA on ROS production in Col plants. The results indicated that exogenous cytokinin could promote ROS generation under the condition of salt treatment (Supplementary Figure S2). To further determine the characteristic of ROS, we examined the contents of hydrogen peroxide H_2_O_2_. As shown in the results, H_2_O_2_ contents were obviously increased in *AtIPT8-OE* plants under the conditions of estradiol-induction and salt treatment (**Figure 5A**). To assess the effect of cytokinin overproduction on ROS-scavenging capacity, the major antioxidant enzymes activities of CAT and SOD were compared between Col and *AtIPT8-OE* plants. As the results, the activities of CAT and SOD increased about 1.8-fold and 2.5-fold, respectively, after salt treatment in Col and *AtIPT8-OE* plant without estradiol induction. However, after estradiol application the activities of CAT and SOD showed only 1.2-fold and 1.7-fold increase in *AtIPT8-OE* plants (**Figures 5B,C**). These results suggested that the weakened performance of *AtIPT8-OE* plants against salt stress was due to elevated ROS production and declined SOD and CAT activities.

### Transcriptomic Analysis on the Effect of Endogenous Cytokinin Overproduction

**FIGURE 6:**
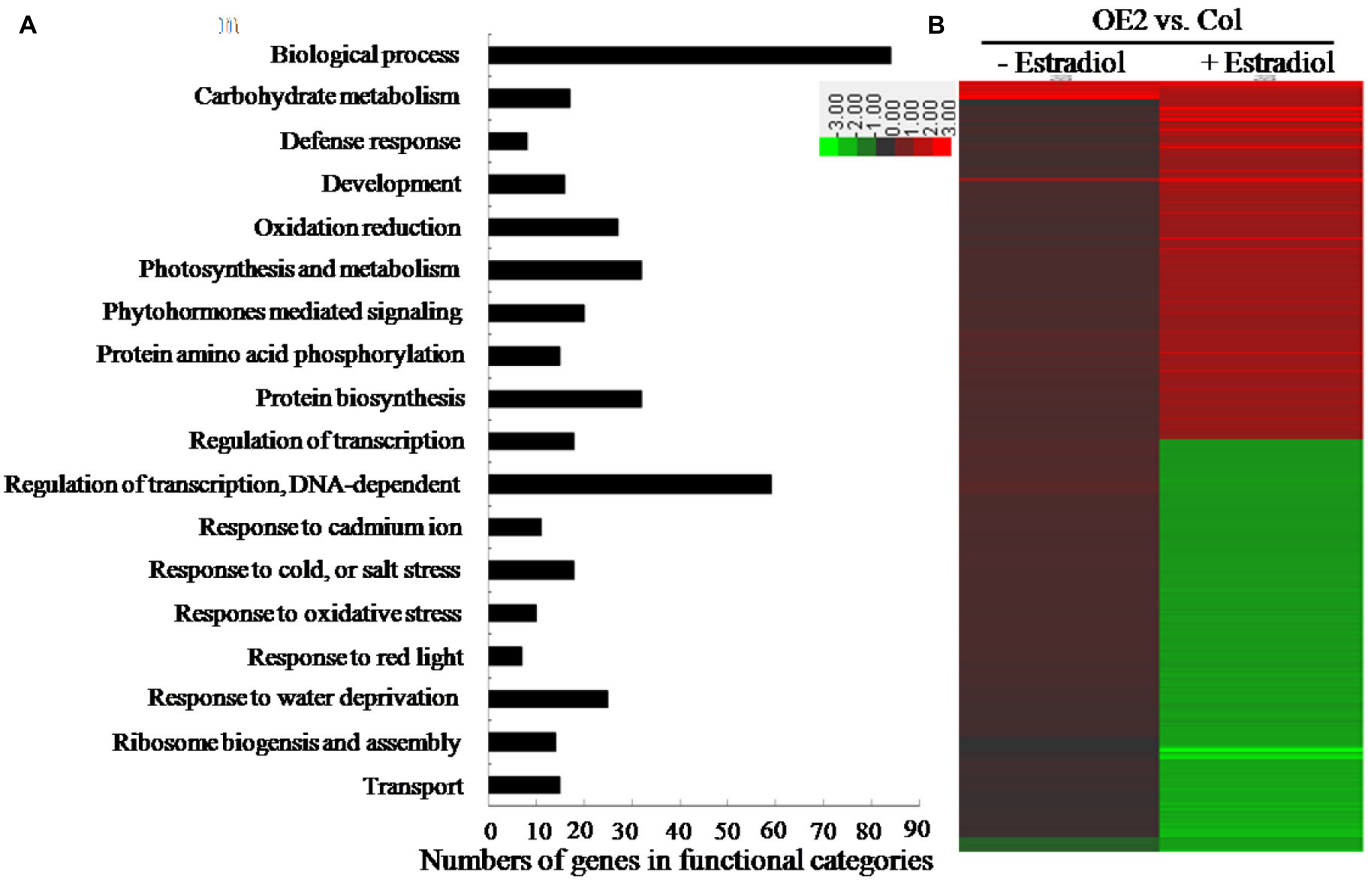
**Differentially expressed genes were identified by microarray analysis between Col and *OE2* plants.** The 10-day-old seedlings were pretreated with 17-β-estradiol (10 μM) or DMSO for 24 h, respectively, and then processed for microarray analysis. **(A)** Profiling for the differentially expressed genes using the CapitalBio^^®^^ Molecular Annotation System V4.0 (CB-MAS) functional catalog. Categories of the differentially expressed genes in *OE2* and Col plants after 17-β-estradiol (10 μM) treatment for 24 h. **(B)** Hierarchical cluster analysis of genes regulated by *AtIPT8* overexpression in *Arabidopsis*. The differentially expressed genes in *OE2* plants after 17-β-estradiol (10 μM) induction were imported for cluster analysis by using Cluster 3.0, and the resulting tree figure was displayed using the Java Treeview software. The detailed information of genes was listed in Supplementary Table S2

To assess the transcriptomic changes which might have been affected by endogenous cytokinin overproduction in *AtIPT8-OE* plants, we conducted the microarray analysis to analyze the potential genes with differential expression levels in Col and *AtIPT8-OE* plants. Ten-day-old seedlings were pre-treated with or without estradiol for 24 h, and then total RNA were extracted for microarray analysis. A Two-Way *Arabidopsis* Genome Array (CapitalBio Corp.^4^) was used in this study (Patterson et al., 2006; Wang et al., 2011). Upon estradiol-induction, 425 genes exhibited more than twofold changes in the transcription levels between Col and *AtIPT8-OE* plants (**Figure 6A**; Supplementary Table S2) (Wang et al., 2011). Functional categorization of the differentially expressed genes revealed that cytokinin overproduction affected the expression of many genes involving in biological process, carbohydrate metabolisms, photosynthesis, transcription regulations and abiotic stress responses (**Figure 6A**). Detailed functional categorization indicated that many differentially expressed genes were the members which could be involved in responding to various stresses, such as the defense responses, oxidation reductions, cold, salt and water deprivation responses. Hierarchical cluster analysis on genes regulated by cytokinin overproduction in *Arabidopsis* indicated that 197 genes were up-regulated and 228 genes were down-regulated in *AtIPT8-OE* plant, when compared with Col control after estradiol-induction (**Figure 6B**). Among them, many ABA- and abiotic stress-related genes might be affected by overproducing cytokinin (Wang et al., 2011). To rule out the effect of cytokinin overeproduction in ROS generation/signaling and salt stress response, we compared the differentially expressed genes by cytokinin with salt- and oxidative-regulated genes, which were downloaded from the public microarray data^5^. Interestingly, among 425 differentially expressed genes in *AtIPT8-OE* plants dependent upon estradiol induction (**Figure 6A**; Supplementary Table S2), only 406 genes could be found in the data from the transcriptomic database (Supplementary Figure S3A; Supplementary Table S2). There were 104 genes with significant changes (folds ≥ 2.0) after treated by cytokinin and salt, respectively; and among of them, 40 genes were up-regulated and 64 genes were down-regulated. Forty-two genes were co-regulated by both of cytokinin and oxidative stress, and among of them 32 genes were up-regulated (Supplementary Figures S3B,C). Only 25 genes have been co-regulated by all of the cytokinin overproduction, salt and oxidative stresses treatment (Supplementary Figure S3B).

### Transcriptional Alterations of ROS-scavenging and -production Related-genes by Endogenous Cytokinin Overproduction and Salt Stress

**FIGURE 7:**
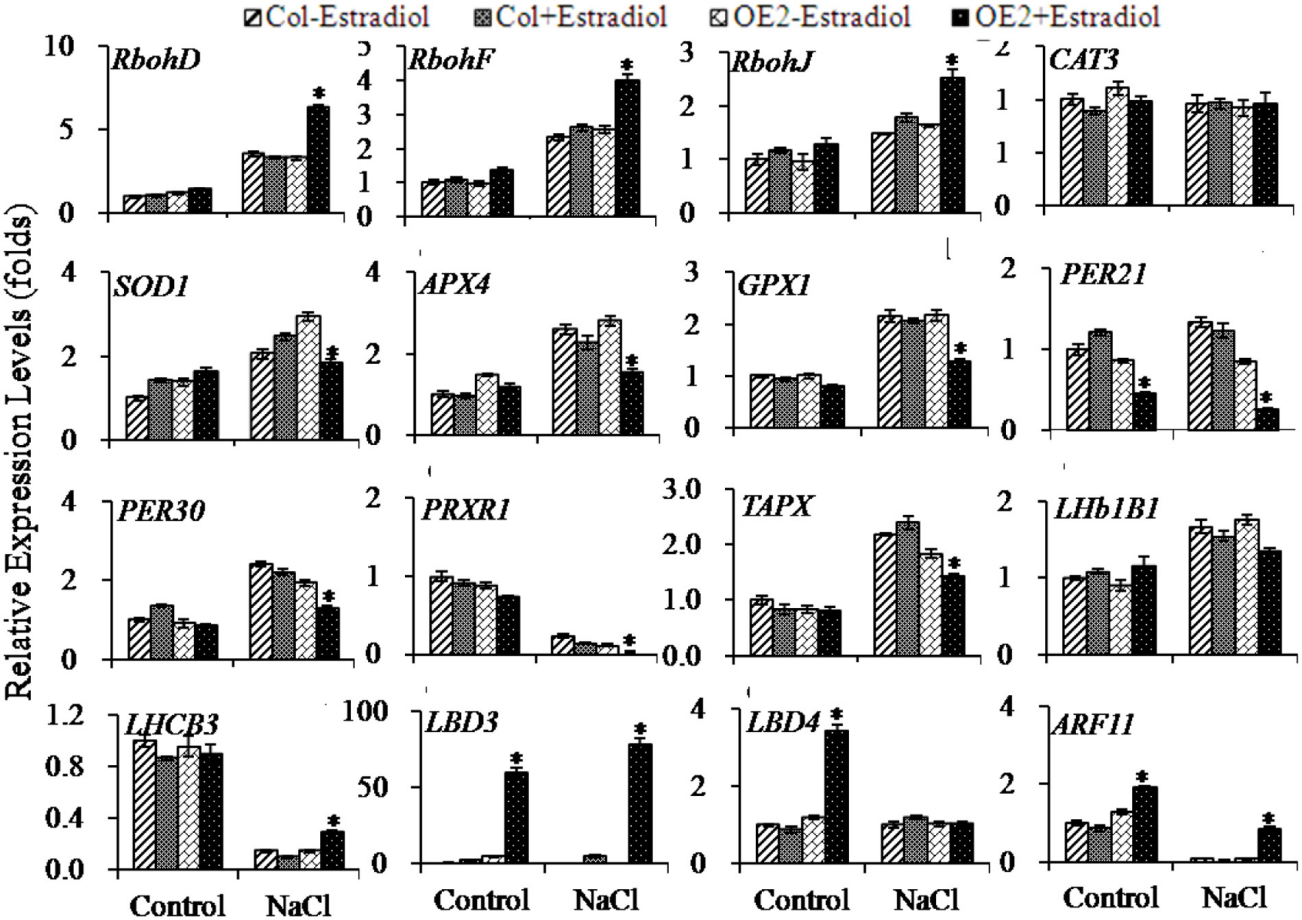
**Quantitative analysis on the expression levels of ROS-related and photosynthesis-related genes.** 10-day-old plants grown on MS plates were pretreated with DMSO or 17-β-estradiol (10 μM) for 24 h, and then subjected to treatments (NaCl, 100 mM; with or without estadiol) in liquid MS medium for 3 h. The seedlings were then collected for total RNA extraction and qRT-PCR analysis. Gene expression levels were normalized to their expression levels in Col plants, which were taken as 1. The data are means ± SE of three independent repeats. Asterisk symbols (^∗^) indicate *p* < 0.05 (Student *t*-test).

Next, we selected some genes which were responsible for ROS-production and -scavenging for follow-up qRT-PCR analyses. Ten-day-old seedlings were pretreated with or without estradiol for 24 h, and then treated with NaCl for 3 h. Because *RbohD*, *RbohF*, and *RbohJ* are responsible for fine tuning the control of ROS production, we attested their expression levels. As shown in the results, three examined *Rboh* genes could be up-regulated by NaCl-treatment either in Col or in *AtIPT8-OE* plants; the significantly enhanced expression levels of these three *Rboh* genes were scored with *AtIPT8-OE* plants upon estradiol-induction and salt treatment (**Figure 7**).

The ROS-scavenging related-genes were also compared between Col and *AtIPT8-OE* plants. Under the condition of salt treatment, the promoted expression levels of *SOD1*, *APX4* and *GPX1* were measured in Col and *AtIPT8-OE* plants while estradiol was present or absent. The expression levels of *SOD1*, *APX4* and *GPX1* were obviously lower in *AtIPT8-OE* plants when compared with those in Col under salt treatment (**Figure 7**). Interestingly, the *CAT3* expression in both of Col and *AtIPT8-OE* plants was not affected in all tested conditions. We also compared the expression levels of some genes encoding peroxidases. As shown in **Figure 7**, upon estradiol-induction the expression levels of *PER21* and *PER30* were inhibited despite of the salt treatment. Moreover, *PRXR1* and *TAPX*, which are involved in the hydrogen peroxide catabolic and oxidation-reduction processes, were analyzed. The down-regulated *PRXR1* transcripts showed in the salt treatment, and significant decrease in expression levels of *PRXR1* were scored in the *AtIPT8-OE* plants that were induced by estradiol. In contrast, up-regulated *TAPX* expression was detected after salt treatment, but estradiol-induction weakened the *TAPX* expression (**Figure 7**).

In addition, we examined expressions of those genes which are functional in the photosystem (Supplementary Table S2). Thus, expressions of *LHb1B1* and *LHCB3* were compared under the condition with or without salt treatment. We observed that increased *LHb1B1* expression level could be triggered by the salt treatment. Notably, the elevated *LHb1B1* expression level could be retracted in *AtIPT8-OE* plants upon estradiol-induction. As for the expression of *LHCB3*, it was obviously inhibited by the salt treatment in all the examined plants; recovered level of *LHCB3* expression was detected with the estradiol-induction in *AtIPT8-OE* plants (**Figure 7**). Furthermore, we analyzed the expressions of *LBD3* and *LBD4*, known as LOB domain-containing proteins and playing roles in the determination of bilateral symmetry (Shuai et al., 2002). The *LBD3* (ASL9, ASYMMETRIC LEAVES 2 LIKE 9) can be exclusively regulated by the plant hormone cytokinins in a manner of depending on His-Asp phosphorelay signal transduction (Naito et al., 2007). In this study, we found that *LBD3* expression was significantly enhanced in *AtIPT8-OE* plants in the estradiol-dependent manner. When treated with NaCl, the higher level of *LBD3* expression was sustained (**Figure 7**; Supplementary Table S2). *LBD4* showed less than four fold increase in *AtIPT8-OE* plants that were induced by estradiol, but the salt treatment revoked the effect of cytokinin overproduction on *LBD4* expression. We also evaluated the auxin responsive factor *ARF11* that could be down-regulated by cytokinin overproduction (Supplementary Table S2). As shown in **Figure 7**, expression level of *ARF11* was decreased by the salt treatment in Col and *AtIPT8-OE* plants; however, slight rebound of *ARF11* expression was produced with estradiol-induction.

## Discussion

Maintaining cytokinin homeostasis is essential for plant growth and development, as well as plant adaptation to environmental stresses. Numerous studies demonstrate that abiotic stresses have both positive and negative effects on the metabolism of endogenous cytokinins (Hansen and Dörﬄing, 2003; Kudoyarova et al., 2007; Alvarez et al., 2008). It is usually difficult to define the working concentrations in plant cells for exogenous application of cytokinins. In this study, through analyzing inducible-*AtIPT8* overexpression transgenic plants we investigated the effects of modulating endogenous cytokinin production in salt treatments. Results in this study demonstrated that inducible *AtIPT8* overexpression could significantly promote endogenous cytokinin overproduction, and affect the responses of *Arabidopsis* plants to salt stresses. The balance of endogenous cytokinin and auxin contents is critical for maintaining primary root growth (Dello Ioio et al., 2007; Müller and Sheen, 2008; Moubayidin et al., 2010; De Rybel et al., 2014; Schaller et al., 2015). In *AtIPT8-OE* plants, the growth of primary roots was significantly inhibited by overproduction of endogenous cytokinin upon estradiol-induction (**Figures 1** and **2**). We further assessed the effect of cytokinin overproduction on osmotic and salt stress responses. Our results indicated that both of salt and osmotic stress treatments inhibited plant leaf and root growth (**Figure 2**). Notably, the most significance in inhibitory effect was observed after glucose treatment, which resulted in an extremely shorten roots and etiolated cotyledons (**Figure 2**). Salt and osmotic stresses have similar effects on water potential, but salinity has additional cytotoxic effects within the cell (Zhu, 2002). When exposed to high salt concentrations, the *AtIPT8-OE* plants showed less survival rates and the chlorophyll contents were significantly decreased after estradiol application (**Figure 3**). It has been shown that, under normal conditions, exogenous cytokinin (6-benzyladenine) application is able to promote chlorophyll biosynthesis in roots, but, mutations in cytokinins receptors (*ahk2-2ahk3-3* and *cre1-12ahk3-3*) result in lower chlorophyll contents (Kobayashi et al., 2012). In this study, we observed that, if only overproduced endogenous cytokinin in *AtIPT8-OE* plants it had no obvious effects on chlorophyll contents (**Figure 3**). It is likely, the regulations of cytokinins and chlorophyll biosynthesis is much more complicated than we would have expected. Future studies on this point will expand our understanding on the complications of cytokinins and chlorophyll biosynthesis in *Arabidopsis*.

Chlorophyll accumulation is important in abiotic stress responses, because plant cells must strictly regulate their metabolisms to coincide with the machinery of photosynthesis (Tanaka et al., 2011). Interestingly, in our results, we have noticed that many genes, which are involved in the photosynthesis and metabolism, were differentially expressed in the *AtIPT8-OE* plants that were overproducing endogenous cytokinins (**Figures 6** and **7**, Supplementary Table S2). For instance, genes encoding the components of light harvesting protein complexes, such as LHb1B1, LHCB2.2, LHCB3, and LHCB4 were differentially regulated by overproduced endogenous cytokinins and the salt treatment (**Figure 7**; Supplementary Table S2). Expression levels of the photosystem II subunits including PSAK, PSAN, PSBP, and PSBQ, which are involved in oxygen evolution, were down-regulated by cytokinin overproduction (Supplementary Table S2). Nowadays, fewer evidences in the involvement of photosystem II subunits in abiotic stress responses are reported. With altered functions of chlorophyll-binding proteins, the sensitivity of ABA and dehydration conditions may be influenced in plants (Xu et al., 2012). Our results in analyzing the chlorophyll contents and in profiling the photosystem related genes suggested an indispensable mechanism that may involve in modulating endogenous cytokinin levels and responding to abiotic stress conditions.

The expression levels of stress-responsive genes that can be altered at various degrees after cytokinin treatment were revealed by genome-wide transcriptome analyses (Brenner et al., 2012; Bhargava et al., 2013; Brenner and Schmülling, 2015). The effects of salt stress and cytokinin-deficiency on gene expression have been demonstrated, in which a subset of stress-responsive genes are significantly modified in the cytokinin-deficient mutant *ipt1,3,5,7*, under normal and salinity conditions (Nishiyama et al., 2012). Under salinity conditions, cytokinin-deficiency may up-regulate many stress-responsible genes, including DREB-type transcriptional factors, ABA-responsive components, as well as salt-inducible *NAC* and *ZFHD* genes (Nishiyama et al., 2012). In agreement with this trend, we demonstrated that cytokinin-overproduction inhibited ABA-signaling downstream targets such as *ABF3*, *RAB18*, *RD29B*, *RD26*, *DREB2A*, as well as homeobox proteins *ATHB5*, *ATHB7*, and *ATHB12* (Supplementary Table S2). Thus, cytokinin and ABA are functionally antagonized in the regulation of plant growth and the adaption of abiotic stresses (Shkolnik-Inbar and Bar-Zvi, 2010; Nishiyama et al., 2011; Wang et al., 2011; Liu et al., 2013; Guan et al., 2014; Yang et al., 2014).

In general, abiotic stress triggers oxidative responses and then stimulates ROS production. In this study, many of the differentially expressed genes, which were triggered by the overproduction of endogenous cytokinins, could be categorized into oxidation reduction and oxidative stress responses (**Figure 6**; Supplementary Table S2). Endogenous cytokinin overproduction enhanced ROS generation and decreased the activities of ROS-scavenging enzymes (**Figures 4** and **5**). In plant cells, ROS production occurs mainly in membrane-enclosed compartments such as chloroplasts, mitochondria and peroxisomes. In chloroplasts, photosystem I and II (PSI and PSII) are the major sites for ROS generation. Emerging evidences have implicated that cytokinin signaling in abiotic stresses lead to photosynthetic dysfunction and ROS production, by affecting genes expression of PSII subunits (Yi et al., 2008; Kobayashi et al., 2012). The enhancement of expressions of *RbohD*, *RbohF*, and *RbohJ* genes, which was triggered by the salt treatment and cytokinin overproduction, suggested the complex network of cytokinin, salt stress and ROS generation in plant cells (**Figure 7**). Overexpression of ROS-scavenging enzymes, such as isoforms of SOD, CAT and APX, can stimulate abiotic stress tolerance in various crop plants (Apel and Hirt, 2004). In this study, expressions of *SOD1*, *APX4*, *GPX1*, *PER21*, *PER30*, *PXRX1*, and *TAPX1* were significantly down-regulated by endogenous cytokinin-overproduction and salt-treatment (**Figure 7**; Supplementary Table S2). In contrast to the complex effects of cytokinin homeostasis to drought stress tolerance, cytokinin deficient mutant *ipt1,3,5,7* resists to salt stress (Nishiyama et al., 2011). Notably, in agreement with this study, we showed that overproduction cytokinin could enhance salt sensitivity in *Arabidopsis*. Thus, under the conditions of endogenous cytokinin overproduction and salt treatment, it is likely that, the lower expression levels of ROS-scavenging related-genes and the promotion of ROS-production were attributed to the decrease in antioxidant enzyme activities and the increase in ROS contents in *AtIPT8-OE* plants.

*LBD*s mainly expressed at the base of lateral organs of shoots and roots. Ectopically overexpressing *LBD* results in smaller organs through limiting the cell division (Shuai et al., 2002). Previous studies indicate that cytokinin is crucial for determining root-meristem size and root stem-cell specification (Dello Ioio et al., 2007; Müller and Sheen, 2008). In this study, significantly up-regulated *LBD3* was linked to the overproduction of endogenous cytokinins and the treatment of salt, which was consistent with a previous study (Naito et al., 2007). Not like *LBD3*, the expression level of *LBD4* was slightly increased by overly produced endogenous cytokinins, and the salt treatment antagonized the effect of cytokinin on *LBD4* expression (**Figure 7**; Supplementary Table S2). The pleiotropic defects in the growth of roots and cotyledons, caused by endogenous cytokinin overproduction, might be achieved by enhancing *LBD*s expression. Collectively, we concluded that endogenous cytokinin overproduction derived by inducible overexpression of *AtIPT8* shed a negative effect on plant salt tolerance by modulating stress-responsive gene expression, ROS production and chlorophyll homeostasis.

## Author Contributions

In this research, YW was responsible for the experimental design, revising and finalizing the manuscript. YW designed and performed most of the experiments, analyzed the data and drafted the manuscript. WS performed physiological, confocal microscopic imaging and gene expression experiments. ZC provided regents and helpful discussions. All the authors in this research read and approved the final manuscript.

## Conflict of Interest Statement

The authors declare that the research was conducted in the absence of any commercial or financial relationships that could be construed as a potential conflict of interest.

## Footnotes

1 http://tiangen.biomart.cn

2 http://www.toyobo-global.com

3 https://www.genscript.com/ssl-bin/app/primer

4 http://www.capitalbio.com

5 http://bar.utoronto.ca/welcome.htm

## Supplementary material

FIGURE S1 **The inhibited elongation of primary roots in *OE2* can be rescued by IAA treatment. (A)** Exogenously added IAA could rescue estradiol-induced aberrant growth of roots in *OE2*. Seeds were growing on MS plates containing 17-β-estradiol (10μM) for 2 weeks, then transferred to medium containing IAA (1.0 nM) for 5 days. (Bar = 5cm). **(B)** The primary root lengths (shown in **A**) were measured. The results represent the means + SE of three independent experiments (seedling numbers, *n* > 30 per experiment). **(C)** 17-β-estradiol (10 μM) treatment could induce more callus generation in *OE2* plants than that in Col under the same concentrations of exogenous 2,4-D (5 nM). Photos were taken after 7 days growth on MS plates.

FIGURE S2 **Exogenous application of cytokinin (6-BA) enhanced ROS production under the condition of salt stress. (A)** The 5-day-old Col seedlings were pretreated with NaCl (100 mM) plus or minus 6-BA (10 μM) for 3 days. The fluorescent intensity of DCF was observed using the confocal microscope. Bar, 50 μm. **(B)** The ROS contents in Col plants were quantified. The data are means ± SEs of three independent experiments (*n* = 10). The fluorescent intensity of cotyledons in Col without salt treatment was taken as “1.”

FIGURE S3 **Comparisons of differentially expressed genes in *AtIPT8-OE* plants and salt- and/or oxidative-stress regulated genes downloaded from public microarray database (http://bar.utoronto.ca/welcome.htm). (A)** Hierarchical cluster analysis of genes affected by *AtIPT8* overexpression, salt and oxidative stresses in *Arabidopsis*. The differentially expressed genes in *OE2* plants after 17-β-estradiol (10 μM) induction, and by the salt and oxidative treatments were analyzed by the method of using Cluster 3.0. The resulting tree figure was displayed using the Java Treeview software. The detailed information of genes was listed in Supplementary Table S2. **(B)** Numbers of overlapping transcripts changed by cytokinin overproduction, salt and oxidative treatments. **(C)** Numbers of genes with up- or down-regulated expression levels by cytokinin overproduction, salt and oxidative treatments.

TABLE S1 **Primers used for qRT-PCR analysis in this study**.

TABLE S2 **The differentially expressed genes in *AtIPT8-OE plant* and ROS-regulated and/or salt-regulated genes from public microarry data (http://bar.utoronto.ca/welcome.htm)**.
