# Supplementary figures and images for: Endogenous Cytokinin Overproduction Modulates ROS Homeostasis and Decreases Salt Stress Resistance in Arabidopsis Thaliana
Source: Front Plant Sci. 2015 Nov 19;6:1004. doi: 10.3389/fpls.2015.01004 (PMC4652137; doi:10.3389/fpls.2015.01004)

**Figure S1**

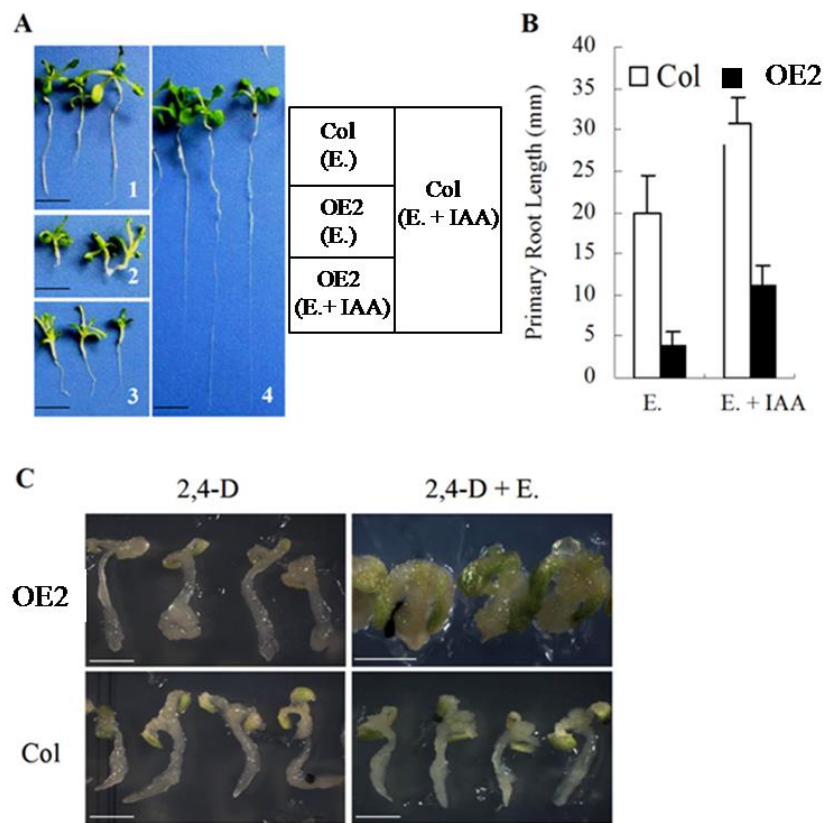

**Figure S2**

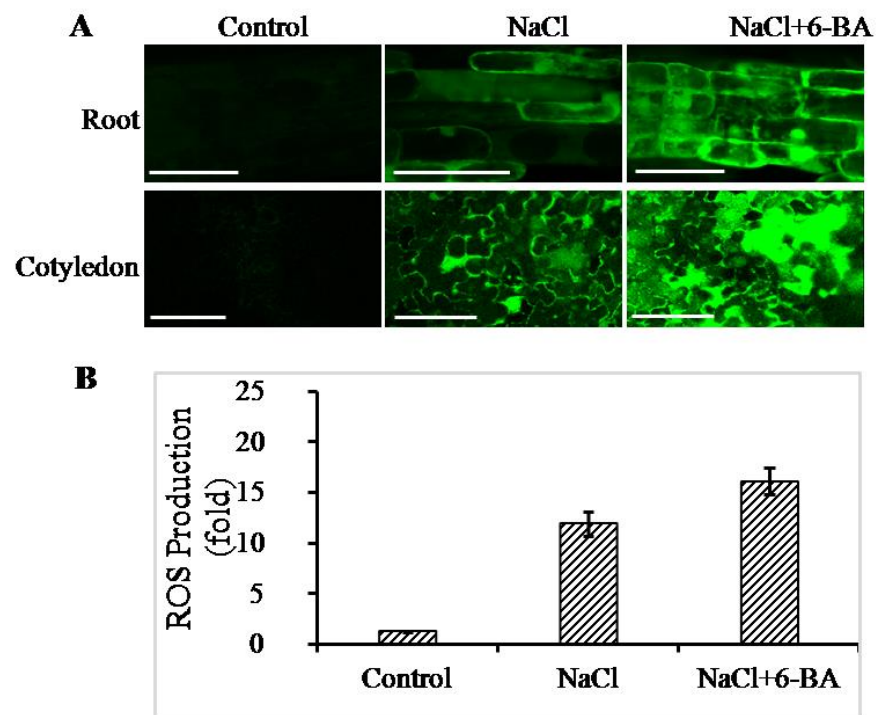

Figure S3

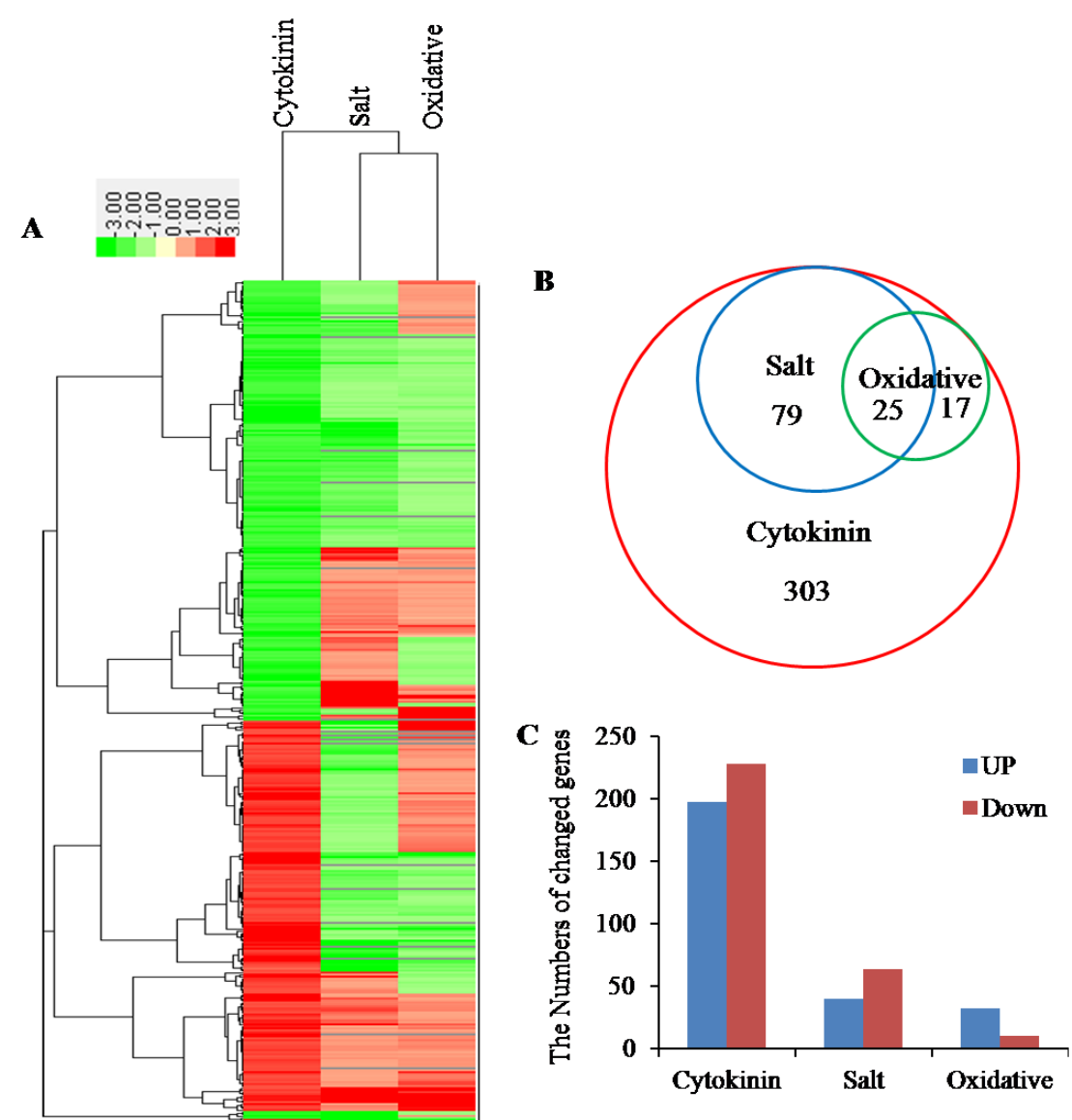

Supplement: FIGURE S1 — The inhibited elongation of primary roots in OE2 can be rescued by IAA treatment. (A) Exogenously added IAA could rescue estradiol-induced aberrant growth of roots in OE2. Seeds were growing on MS plates containing 17-β-estradiol (10μM) for 2 weeks, then transferred to medium containing IAA (1.0 nM) for 5 days. (Bar = 5cm). (B) The primary root lengths (shown in A) were measured. The results represent the means + SE of three independent experiments (seedling numbers, n > 30 per experiment). (C) 17-β-estradiol (10 μM) treatment could induce more callus generation in OE2 plants than that in Col under the same concentrations of exogenous 2,4-D (5 nM). Photos were taken after 7 days growth on MS plates. [file Data_Sheet_1.PDF]
